# Proteomic identification of MYC2-dependent jasmonate-regulated proteins in *Arabidopsis thaliana*

**DOI:** 10.1186/1477-5956-10-57

**Published:** 2012-09-25

**Authors:** Jing Guo, Qiuying Pang, Lihua Wang, Ping Yu, Nan Li, Xiufeng Yan

**Affiliations:** 1 College of Life and Environmental Science, Wenzhou University, Wenzhou, 325035, China; 2 Alkali Soil Natural Environmental Science Center; Key Laboratory of Saline-alkali Vegetation Ecology Restoration in Oil Field, Ministry of Education, Northeast Forestry University, Harbin, 150040, China

**Keywords:** *Arabidopsis thaliana*, Jasmonate, MYC2, Proteomics

## Abstract

**Background:**

MYC2, a basic helix-loop-helix (bHLH) domain-containing transcription factor, participates in the jasmonate (JA) signaling pathway and is involved in the modulation of diverse JA functions. However, a comprehensive list of MYC2-dependent JA-responsive proteins has yet to be defined.

**Results:**

In this paper, we report the comparative proteomics of wild-type (WT) plants and *jin1-9*, a MYC2 mutant plant, in response to methyl jasmonate (MeJA) treatment. Proteins from mock/MeJA-treated *jin1-9* and WT samples were extracted and separated by two-dimensional gel electrophoresis. Twenty-seven JA-mediated proteins demonstrated differential expression modulated by MYC2. We observed that MYC2 negatively regulates the accumulation of JA-dependent indolic glucosinolate-related proteins and exhibits opposite effects on the biosynthetic enzymes involved aliphatic glucosinolate pathways. In addition, proteins involved in the tricarboxylic acid cycle and a majority of the MeJA-inducible proteins that are involved in multiple protective systems against oxidative stress were reduced in *jin1-9/myc2* sample compared to the WT sample. These results support a positive role for MYC2 in regulating JA-mediated carbohydrate metabolism and oxidative stress tolerance.

**Conclusions:**

We have identified MYC2-dependent jasmonate-regulated proteins in *Arabidopsis thaliana* by performing two-dimensional gel electrophoresis and MALDI-TOF/TOF MS analysis. The observed pattern of protein expression suggests that MYC2 has opposite effects on the biosynthetic enzymes of indolic and aliphatic glucosinolate pathways and positively regulates JA-mediated carbohydrate metabolism and oxidative stress tolerance-related proteins. Furthermore, it is very interesting to note that MYC2 plays opposite roles in the modulation of a subset of JA-regulated photosynthetic proteins during short-term and long-term JA signaling. This study will enhance our understanding of the function of MYC2 in JA signaling in *Arabidopsis thaliana*.

## Introduction

Jasmonates (JAs), including jasmonate and methyl jasmonate (MeJA), are a group of plant growth regulators and signaling molecules derived from linolenic acid. They are well known because they have pivotal roles in many aspects of plant development such as root growth, senescence, fertility, production of secondary metabolites and responses to abiotic and biotic stresses [1-5].

To date, various genes involved in the JA signaling pathway have been identified. The *CORONATINE-INSENSITIVE1* (*COI1*) gene, which is a JA receptor, encodes an F-box protein [6,7]. COI1 combines with Skp1-like 1, Skp1-like 2, cullin 1, and ring-box protein 1 to form an E3 ubiquitin ligase known as the SCF^COI1^ complex [8-10], which promotes the ubiquitination and degradation of the repressors of the JA signaling pathway. The JA ZIM-domain (JAZ) proteins (JAZ1 to JAZ12) that share a conserved ZIM and Jas motif are targets of the SCF^COI1^ complex; therefore, they act as negative regulators of the JA-responsive genes [11-16]. Recently, it was demonstrated that a co-receptor complex consisting of COI1, JAZ, and inositol pentakisphosphate strongly binds to (+)-7-iso-JA-Ile and therefore functions as the jasmonate receptor [17]. In addition, three closely related basic helix-loop-helix (bHLH) domain-containing transcription factors (TFs), MYC2, MYC3, and MYC4, interact with JAZs to mediate JA responses [18-20].

MYC2 is the most well-known and studied MYC isoform and is involved in JA signaling. *MYC2* is allelic to the *JAI1*/*JIN1* (*JASMONATE-INSENSITIVE1*) locus and binds to the G-box and derived sequences in the promoter regions of JA-induced genes [11,21-23]. All three MYC proteins showed highest binding affinity to a canonical G-box, whereas MYC2 and MYC3 were undistinguishable, MYC4 showed lower affinity for the G-box variants [19]. It has been established that MYC3 and MYC4 act in concert with MYC2 during the activation of JA responses, including JA-induced expression of *VSP2*[19]. In a recent study, by performing microarray analysis on the *jin1* mutant, it was demonstrated that MYC2 regulates the expression of a considerable number of JA-responsive genes, including the genes involved in JA-induced indolic glucosinolate and auxin biosynthesis, and those related to oxidative stress tolerance, and insect herbivory resistance [22]. However, the proteins regulated by MYC2 at the protein level remain to be elucidated.

In the present study, the effect of MeJA on the *jin1-9/myc2* and WT *Arabidopsis thaliana* (Arabidopsis) proteome was assessed by two-dimensional gel electrophoresis (2-DE) and MS/MS analysis. The main objectives of this study were to identify proteins that were differentially expressed in the mock and MeJA-treated *jin1-9/myc2* and WT samples and to elucidate the role of MYC2 in JA signaling pathway. A list of MYC2-dependent JA-responsive proteins was obtained, and this will enhance our understanding of the function of MYC2 in JA signaling in Arabidopsis.

## Results and discussion

### Identification of MYC2-dependent JA-regulated proteins by 2-DE and MS/MS

**Figure 1:**
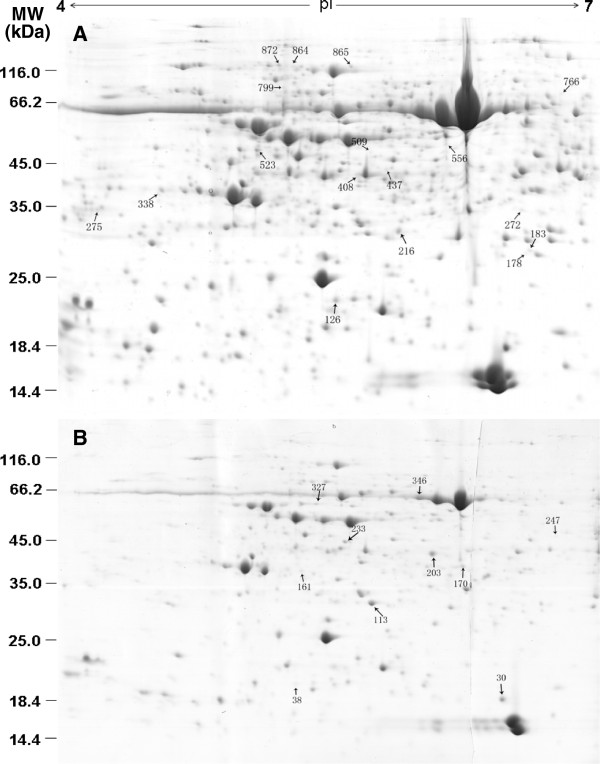
**2-DE gel analysis of proteins extracted from the *jin1-9/myc2 *mutant.** Separation on the first dimension was performed using 1300 μg of total soluble proteins using the linear gradient IPG strips with pH 4–7. In the second dimension, 12.5% SDS-PAGE gels were used, and proteins were visualized using coomassie brilliant blue. Arrows indicate 27 protein spots that changed reproducibly and significantly in MeJA-treated *jin1-9/myc2* mutant plants compared to MeJA-treated wild-type plants. A and B represent 2-DE gels from *jin1-9/myc2* samples treated with 200 mM MeJA for 6 h and 48 h, respectively. 2-DE experiments were repeated 3 times with independent biological replicates.

To investigate the role of MYC2 in JA-regulated gene expression at the posttranscriptional level, three-week-old WT and *jin1-9/myc2* mutant plants were treated with 200 μM MeJA or mock solution, and a 2-DE strategy was used to analyze protein expression changes in response to short-term (6 h) and long-term (48 h) MeJA treatment. The short-term time point was chosen based on previously published Arabidopsis cDNA microarray reports, which demonstrated that the expression of a large number of genes was significantly altered by MeJA-treatment for 6 h [22]. We hypothesized that gene expression at the transcriptional level may not correlate well with that observed at the protein level [24]; therefore, 48 h was chosen for the long-term treatment. Approximately 1500 protein spots were detected by Coomassie Brilliant Blue staining, and approximately 900 protein spots matched across all the gels. Representative images of the 2D gel maps are shown in Figure 1. Proteins were well separated in both dimensions. The percentage volumes of protein spots in triplicate samples were subjected to statistical analysis, and the protein spots were considered to be differentially expressed if they had a relative volume ratio above the threshold (> 1.5 fold and *p* < 0.05) for at least one time point after MeJA treatment. The comparative image analysis of MeJA-treated WT and *jin1-9/myc2* mutant plants led to the identification of 30 protein spots that had changed significantly in abundance (*p* < 0.05). Protein spots that contained differentially expressed proteins were excised, trypsin digested, and analyzed by MS analysis. These 30 protein spots were subjected to MS/MS sequencing via MALDI-TOF mass spectrometry, and 27 unique proteins were successfully identified with high confidence by MS analysis and by performing a search against the MASCOT database (Figure 1, Table 1, Additional file 1). These results suggest that both MeJA treatment and the presence of MYC2 are required for the regulation of these 27 proteins.

**Table 1:** **Protein identities and relative changes in their expression between MeJA-treated *jin1-9/myc2 *and wild-type plants after 6 h or 48 h of MeJA treatment**

**Spot No.**^**a**^	**Category and name**	**gi Number**^**b**^	**Exp pI/kDa**^**c**^	**Thero pI/kDa**^**d**^	**Score**^**e**^	**SC**^**f**^**(%)**	**PN**^**g**^	**MeJA-Treated WT 6h**	**MeJA-Treated jin1-9 6h**^**h**^	**Fold change**	***p-value***	**MeJA-Treated WT 48h**	**MeJA-Treated jin1-9 48h**^**h**^	**Fold change**	***p-value***
								**Vol% (mean±SD)**	**Vol% (mean±SD)**			**Vol% (mean±SD)**	**Vol% (mean±SD)**		
	**Glucosinolate metabolism**														
216	glutathione S-transferase ERD13 (GSTF10)^Neg^	gi|15224582	5.82/33.0	5.54/29.8	66	5	2	0.11±0.00	0.17±0.00	1.51	0.00	0.17±0.10	0.09±0.09	-1.88	0.37
327	myrosinase-binding-like protein	gi|30684083	5.14/76.0	5.12/48.5	131	21	6	0.05±0.01	0.04±0.00	-1.15	0.23	0.08±0.02	0.04±0.01	-2.10	0.02
509	sulfotransferase 18	gi|15221130	5.66/57.0	5.52/40.7	177	13	4	0.01±0.00	0.00±0.00	-9999	0.01	0.00±0.00	0.00±0.00	—	—
523	3-isopropylmalate dehydrogenase^up^	gi|15241338	5.04/58.0	5.75/44.2	88	2	1	0.08±0.00	0.05±0.01	-1.63	0.00	0.00±0.00	0.00±0.00	—	—
556	amidase 1	gi|18390964	6.08/61.0	5.83/45.4	152	22	6	0.02±0.01	0.00±0.00	-9999	0.04	0.02±0.03	0.00±0.00	-9999	0.19
864	thioglucoside glucohydrolase 1 (TGG1)	gi|15809938	5.24/121.0	5.67/61.7	141	10	4	0.02±0.01	0.00±0.00	-9999	0.02	0.00±0.00	0.00±0.00	—	—
	**Stress and defense**														
113	glutathione S-transferase DHAR1^Pos^	gi|8778432	5.66/31.9	6.82/50.6	150	6	2	0.12±0.03	0.07±0.02	-1.75	0.05	0.48±0.42	0.31±0.02	-1.56	0.00
161	putative glyoxalase	gi|9828630	5.08/38.9	6.97/40.3	85	12	3	0.04±0.02	0.00±0.00	-9999	0.02	0.05±0.01	0.02±0.01	-2.22	0.01
183	glutathione S-transferase (AtGSTF3)	gi|497788	6.40/29.1	5.80/23.5	116	11	2	0.03±0.01	0.00±0.00	-9999	0.00	0.08±0.00	0.01±0.00	-14.16	0.00
203	putative arginase^Pos^	gi|15236635	6.01/44.0	5.90/38.1	163	12	4	0.11±0.01	0.13±0.01	1.14	0.05	0.30±0.02	0.19±0.03	-1.61	0.01
275	HSP20-like chaperones protein	gi|3193303	4.17/36.0	4.42/28.0	103	16	4	0.04±0.01	0.00±0.00	-9999	0.00	0.02±0.01	0.00±0.00	-9999	0.09
338	putative plastid-lipid-associated protein 1	gi|15233357	4.52/41.0	5.45/35.0	161	23	5	0.02±0.01	0.00±0.00	-9999	0.00	0.13±0.08	0.03±0.03	-4.55	0.09
408	O-acetylserine (thiol) lyase B (oasB)	gi|15224351	5.59/46.0	8.13/42.0	183	29	8	0.02±0.00	0.10±0.01	4.70	0.00	0.02±0.01	0.00±0.00	-9999	0.12
437	O-acetylserine (thiol) lyase isoform C (oasC)	gi|6899947	5.75/49.0	6.96/41.5	96	12	3	0.02±0.01	0.00±0.00	-9999	0.01	0.00±0.00	0.00±0.00	—	—
872	luminal binding protein (BiP2)	gi|1303695	5.16/122.0	5.08/73.7	165	11	6	0.04±0.01	0.03±0.00	-1.67	0.04	0.00±0.00	0.00±0.00	—	—
	**Photosynthesis**														
126	chlorophyll a/b-binding protein	gi|13265501	5.47/23.9	6.52/26.1	178	9	3	0.12±0.01	0.19±0.01	1.60	0.00	0.07±0.00	0.06±0.00	-1.08	0.13
170	chloroplast NAD-MDH	gi|3256066	6.18/39.8	8.48/42.6	295	14	5	0.17±0.11	0.03±0.03	-5.88	0.10	0.13±0.01	0.09±0.02	-1.55	0.03
233	coproporphyrinogen III oxidase	gi|240254000	5.51/50.0	6.24/44.1	155	35	9	0.07±0.00	0.07±0.00	-1.02	0.14	0.10±0.01	0.06±0.00	-1.62	0.01
247	uroporphyrinogen decarboxylase 2	gi|15226690	6.72/54.0	8.29/43.7	517	32	11	0.07±0.00	0.07±0.00	-1.05	0.10	0.03±0.01	0.00±0.00	-9999	0.01
346	ribulose bisphosphate carboxylase	gi|1944432	5.93/81.0	6.12/48.0	167	16	7	1.04±0.68	0.13±0.09	-8.33	0.08	1.00±0.26	0.00±0.00	-9999	0.00
865	transketolase-like protein	gi|7329685	5.56/120.0	5.80/81.9	113	17	8	0.08±0.00	0.15±0.02	1.84	0.01	0.00±0.00	0.00±0.00	—	—
	**Carbohydrate metabolism**														
272	3-oxoacyl-[acyl-carrier protein] reductase (NADH)	gi|15229203	6.40/36.1	6.12/28.2	61	9	2	0.03±0.01	0.00±0.00	-9999	0.00	0.03±0.00	0.03±0.00	-1.31	0.05
766	2-oxoglutarate dehydrogenase, E3 subunit	gi|4210334	6.40/95.0	6.96/54.0	137	34	5	0.02±0.00	0.00±0.00	-9999	0.00	0.00±0.00	0.00±0.00	—	—
	**Protein synthesis, folding and destination**														
38	40S ribosomal protein S12-1	gi|15218373	5.05/20.0	5.38/15.7	73	22	2	0.08±0.01	0.02±0.00	-3.76	0.00	0.08±0.01	0.03±0.01	-2.37	0.01
178	ATP-dependent Clp protease proteolytic subunit 2	gi|18420643	6.40/29.1	6.71/26.4	92	14	3	0.02±0.01	0.00±0.00	-9999	0.01	0.00±0.00	0.00±0.00	—	—
799	TCP-1/cpn60 chaperonin family protein	gi|15231255	5.19/101.0	5.60/63.7	308	27	12	0.01±0.01	0.00±0.00	-9999	0.02	0.00±0.00	0.00±0.00	—	—
	**other**														
30	SRPBCC ligand-binding domain-containing protein	gi|15236566	6.41/18.1	5.91/17.6	72	15	2	0.12±0.08	0.11±0.06	-1.08	0.89	0.34±0.03	0.00±0.00	-9999	0.00

^a^ Assigned spot number as indicated in Figure 1. ^b^ Database accession numbers according to NCBInr. ^c^ Experimental mass (kDa) and pI of identified proteins. ^d^ Theoretical mass (kDa) and pI of identified proteins.^e^ Mascot score reported after searching against the NCBInr database. ^f^ Sequence coverage. ^g^ Number of peptides sequenced. ^h^ Spot abundance change is expressed as the ratio of intensities (vol.%) of up-regulated (positive value) or down-regulated (negative value) proteins between MeJA-treated *jin1-9* and WT. ^Neg^ Negatively regulated by MYC2. ^Pos^ Positively regulated by MYC2. ^up^ Up-regulated by MeJA. Paired Student's t-test was used to define the significant changes ( *p* <0.05). -9999 indicates the protein spots were not detectable in *jin1-9* after MeJA treatment, short string indicates the protein spots were not detectable in WT and *jin1-9* after MeJA treatment. Three treatments were performed.

To confirm whether MeJA is indispensable for the regulation of these 27 proteins, protein profiles of WT and *jin1-9/myc2* mutant plants that were mock-treated were compared. Our results demonstrate that, in the absence of MeJA treatment, there are no obvious differences in the expression of these 27 proteins (< 1.5 fold or *p* > 0.05) between the WT and *jin1-9/myc2* mutant plants (Additional file 2). These results suggest that MeJA treatment is essential for the regulation of these proteins. To verify whether MYC2 is required for JA-regulated expression of these 27 proteins, the protein profiles of mock-treated *jin1-9/myc2* and MeJA-treated *jin1-9/myc2* mutant plants were analyzed, and no significant differences (< 1.5 fold or *p* > 0.05) were observed (Additional file 2). Thus, MYC2 is essential for the varied expression of these proteins, and JA regulates the expression of these 27 proteins in a MYC2-dependent manner. As shown in Table 1, after 6 h of MeJA treatment, the expression of 19 proteins in *jin1-9/myc2* plants changed significantly. The expression of 15 proteins decreased, whereas the expression of 4 proteins increased. The expression of 11 proteins was significantly decreased in *jin1-9/myc2* plants that were treated with MeJA for 48 h. These results suggest that MYC2 might predominantly play a positive role in short-term and long-term JA signaling events.

### Functional classification of JA-regulated proteins

**Figure 2:**
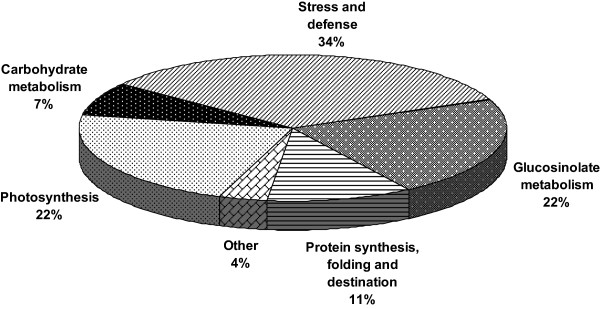
**Classification of the identified proteins based on their molecular functions.** The pie chart indicates the percent distribution of the MYC2-dependent JA-regulated proteins into their functional classes.

Identification of proteins that are differentially expressed due to MeJA treatment and MYC2 expression is an important step in understanding JA-regulated MYC2-dependent signaling pathways. JA plays a critical role in plant defense against pathogens and insects and modulates the biosynthesis of secondary metabolites, the development of the flower and fertility by regulating the expression of related genes [25]. To further examine the differentially expressed proteins, the identified proteins were grouped according to functional categories based on Gene Ontology and by using the MAPMAN software application. As expected, the identified proteins covered a wide range of molecular functions, including glucosinolate metabolism (22.2%), stress and defense (33.3%), photosynthesis (22.2%), carbohydrate metabolism (7.4%), protein folding and degradation (11.1%), and others (3.7%) (Table 1, Figure 2). The three largest groups of proteins consisted of 21 proteins, which were associated with photosynthesis, stress and defense, and glucosinolate metabolism.

### Glucosinolate metabolism

Glucosinolates are a group of nitrogen- and sulfur-containing secondary metabolites that largely exist in the Brassicaceae plants, including Brassica crops and the reference plant, Arabidopsis [26,27]. Glucosinolates are biosynthesized from amino acids and are classified as aliphatic, aromatic, and indolic glucosinolates depending on the origin of the amino acid. The biosynthesis of glucosinolates occurs in three stages, including side-chain elongation of the amino acids, formation of the core structure, and secondary modifications [28], and it is regulated by the JA signaling pathway [29-31].

An increase in glutathione S-transferase ERD13 (GSTF10, spot 216), a protein involved in indolic glucosinolate biosynthesis, was detected in *jin1-9/myc2* compared to WT plants after 6 h of MeJA treatment (Table 1). GSTF10 belongs to the phi class of GSTs and catalyze the biosynthesis of the glucosinolate core structure by adding cysteine during CYP83-catalyzed aldoxime oxidation to form s-alkyl-thiohydroximate in indolic glucosinolate biosysthesis [32,33]. In a previous study, microarray analysis was used to demonstrate that MYC2 negatively regulates JA-dependent indolic glucosinolate biosynthesis [22]. In addition, previous studies showed that *GSTF10* was induced by JA/MeJA, and at the mRNA level, the induction was stronger with MeJA in *jin1-9/myc2* compared to WT plants [22,34]. Consistent with these observations, our results indicate that GSTF10 is negatively regulated by MYC2 in response to MeJA treatment. Within the indolic glucosinolate biosynthetic pathway, the indole-3-acetaldoxime pathway overlaps with the IAA biosynthetic pathway in indolic glucosinolate-producing plant species [35]. It has been reported that MeJA increases IAA levels [22,36]. Our result provide additional proof to support the observation that amidase 1 (AMI1, spot 556), a specific indole-3-acetamide amidohydrolase that catalyzes the synthesis of indole-3-acetic acid from indole-3-acetamide [37], is induced in response to MeJA treatment, and the expression of AMI1 is decreased in *jin1-9/myc2* plants compared to WT plants after treatment with MeJA for 6 h. This result indicates that MYC2 is involved in MeJA-induced IAA biosynthesis and might coordinate MeJA-induced IAA and indolic glucosinolate biosynthetic pathways.

As to the proteins involved in aliphatic glucosinolate biosynthesis, we observed that, following MeJA treatment for 6 h, sulfotransferase 18 (SOT18, spot 509) and 3-isopropylmalate dehydrogenase (IPMDH1, spot 523) were expressed at reduced levels in *jin1-9/myc2* plants compared to WT plants (Table 1). Sulfotransferases catalyze the sulfation of desulfoglucosinolates, the final step in the biosynthesis of the glucosinolate core structure [38]. In Arabidopsis, three sulfotransferases catalyze a broad range of sulfation reactions for desulfoglucosinolates; however, in a competitive situation, SOT18 (AtST5b) preferentially catalyzes the formation of long-chain desulfoglucosinolates derived from methionine [38]. *AtST5b* has been shown to be induced by JA/MeJA [34], and we speculate that JA signaling might regulate aliphatic glucosinolate biosynthesis through AtST5b in a MYC2-dependent manner. IPMDH is involved in leucine biosynthesis and catalyzes NAD^+^-dependent oxidation and decarboxylation of 3-isopropylmalate to produce 4-methyl-2-oxovalerate [39]. The Arabidopsis genome encodes three IPMDHs (AtIPMDH1, AtIPMDH2, and AtIPMDH3), and among these, AtIPMDH1 plays a key role in methionine chain-elongation for the synthesis of aliphatic glucosinolates [40]. Consistent with the up-regulation response of the *AtIPMDH1* after MeJA treatment at the transcriptional level [40], our results suggest that AtIPMDH1 is positively regulated by MYC2 in the JA signaling pathway. In contrast to the negative role of MYC2 in JA-dependent indolic glucosinolate biosynthesis [22], it appears that MYC2 exhibits opposite effects on the biosynthetic enzymes of indolic and aliphatic glucosinolate pathways, as suggested by the expression of GSTF10, IPMDH1, and ST5b in this study. These results might be due to the homeostatic control of glucosinolate synthesis and crosstalk between indolic and aliphatic glucosinolate biosynthesis [28,41].

The degradation products of glucosinolates play important roles in mediating the interactions of plants with their biotic and abiotic environment (e.g., defense against insects, phytopathogens and sulphur/nitrogen metabolism), which are mediated by myrosinases (β-thioglucoside glucohydrolase, TGG). Myrosinases are part of a complex enzyme system that includes myrosinase-binding proteins (MBPs) and myrosinase-associated proteins [42,43]. There are multiple isoenzymes of myrosinase in plants [43], and their expression was demonstrated in restricted tissues [42,44]. Capella et al. reported that *COI1* controls *MBP* expression in flowers and dramatically affects myrosinase expression and activity in Arabidopsis [45]. Myrosinase is a COI1-dependent JA-regulated protein, which was demonstrated by 2-DE DIGE analysis [46]. In this study, a decrease in thioglucoside glucohydrolase 1 (TGG1, spot 864) and myrosinase-binding-like protein (spot 327) was observed in *jin1-9/myc2* plants compared to WT plants in response to MeJA treatment for 6 h and 48 h, respectively (Table 1). These results suggest that MYC2 is a positive regulator of the JA-induced expression of these two proteins. Some myrosinases exhibit pronounced substrate specificity towards glucosinolates (eg. crambe myrosinase from *Crambe abyssinica* is highly specific for epi-progoitrin), and the substrate specificity could be affected by associated factors, including the myrosinase binding protein and myrosinase-associated proteins [43,47]. It is possible that the two proteins (spots 864 and 327) are part of the myrosinase system, which targets the functional glucosinolates involved in the MYC2-dependent MeJA-induced defense reaction.

**Figure 3:**
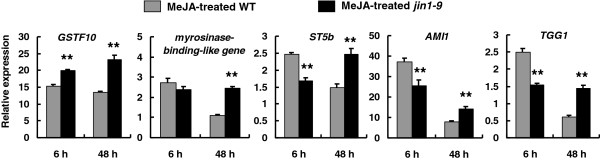
**Quantitative real-time PCR analysis of the transcription of genes encoding proteins identified in 2-DE.** Data are expressed as relative RNA levels ([mRNA]gene/[mRNA]actin) and are the mean of three biological replicates (> 6 pooled plants each); error bars denote SE. Double stars indicate *p* < 0.01 between MeJA-treated *jin1-9/myc2* and wild-type samples.

Expression changes in five genes that encode the identified glucosinolate metabolism-related proteins were analyzed by performing qRT-PCR analysis. Our results demonstrated that the expression of these five genes was similar and consistent at the mRNA and protein level after 6 h of MeJA treatment (Figure 3). However, the expression of these genes at the transcriptional level was different from that observed at the protein level after 48 h of MeJA treatment (Figure 3). This negative protein-mRNA correlation was observed in many other studies [48-50] and indicates that mRNA stability, mRNA splicing, translation, and post-translational events including protein stability and modification are pivotal events in the regulatory mechanisms in biological systems. Thus, proteomic analysis is a direct and efficient approach for studying regulatory mechanisms in biological systems.

### Stress and defense

JA activates plant defense mechanisms in response to biotic and abiotic stresses [51]. It has been reported that *jin1/myc2* plants display increased resistance to fungal and bacterial pathogens [23,52-54]. In the event of biotic stresses, such as herbivory insect feeding, the JA signaling pathway generally accelerates glucosinolate metabolism or the degradation of essential amino acids in the herbivore midgut by activating specific amino acid enzymes [55]. Interestingly, we found that despite the accumulation of the different indolic glucosinolate metabolism-related proteins discussed above, the expression of a putative arginase (spot 203, Table 1), the ortholog of the *Solanum lycopersicum* arginase, which reduces larval weight gain by catabolizing the essential amino acid arginine in the phytophagous insect midgut [55], was reduced in the MeJA-treated *jin1-9/myc2* mutant, which is consistent with the results obtained from transcriptomic analysis [22]. The varied expression of arginase and indolic glucosinolate metabolism-related proteins indicates that MYC2 function is required for JA-mediated insect resistance in Arabidopsis.

JA signaling is important for oxidative stress tolerance. Alteration in the expression of five proteins in response to oxidative stressors was detected, including two cysteine synthases (oasB, spot 408 and oasC, spot 437), two glutathione S-transferases (DHAR1, spot 113 and AtGSTF3, spot 183) and a putative glyoxalase (spot 161) (Table 1). Many abiotic stresses such as drought, heat or salt will cause reactive oxygen species (ROS) production and oxidative damage. In higher plants, glutathione (GSH) plays a considerable role in oxidative stress resistance because it can donate reducing equivalents for scavenging the ROS [56]. Biosynthesis of cysteine is regarded as the rate-limiting step in the production of GSH in plants and is implicated in scavenging ROS and in promoting resistance to abiotic stresses [57,58]. The protein level of cysteine synthetase was induced by MeJA, which produced an increase in the total cysteine content [59,60]. OasB and oasC have been identified as enzymatically true O-acetylserine (thiol) lyases, which catalyze the second step of cysteine biosynthesis [57,61-63]. When *jin1-9/myc2* mutants or WT plants were treated with MeJA for 6 h, the expression of oasB increased, whereas the expression of oasC decreased (Table 1). This indicates that oasB and oasC play diverse roles in instantly responding to oxidative stress in the JA signaling pathway. In addition to cysteine biosynthesis, plants have glyoxalase systems that include glyoxalase I and glyoxalase II, which provide protection against oxidative damage [64], and a peroxisomal membrane-associated ascorbate-dependent electron transfer system, which consists of monodehydroascorbate reductases (MDAR and DHAR) to protect against oxidative stress. Treatment with JA increases the de novo synthesis of ascorbate and DHAR and ascorbate peroxidase activity [3,22,59,65]. We observed that the induction of putative glyoxalase and DHAR1 by MeJA was reduced in *jin1-9/myc2* plants compared to WT plants, and the induction of DHAR1 is consistent with the results obtained from transcriptomic analysis and with the change in dehydroascorbate reductase in the MeJA-treated *coi1-1* mutant [22,46]. These results suggest that JA-induced dehydroascorbate reductase expression is COI-MYC2-dependent. In addition, glutathione S-transferase (AtGSTF3, spot 183), a member of the phi class of GST, which protects plants against oxidative stress [66], is expressed at much lower levels in *jin1-9/myc2* plants compared to WT plants following 6 h and 48 h of MeJA treatment (Table 1). Overall, the expression of majority of the MeJA-induced proteins associated with multiple protective systems against oxidative stress (spots 113, 161, 183, 437) was reduced in *jin1-9/myc2* plants compared to WT plants (Table 1). These results support the positive role of MYC2 in regulating JA-mediated oxidative stress tolerance [22].

Three other proteins were detected in response to stress. A HSP20-like chaperone protein (spot 275) and a fibrillin precursor protein (putative plastid-lipid-associated protein 1, spot 338) were decreased in *jin1-9/myc2* plants compared to WT plants after MeJA treatment (Table 1). HSP20 has a protective role against a variety of stressful conditions, including high temperature, high salt, cold, oxidative stress, water stress and toxic metals [67-73]. Fibrillin accumulation enhances the tolerance of photosystem II toward light stress-triggered photoinhibition in Arabidopsis [74]. Our results demonstrated that HSP20 and the fibrillin precursor protein are positively regulated by MYC2 in the JA signaling pathway and therefore might be associated with MYC2-dependent JA-mediated abiotic stress tolerance. In addition, the expression of a luminal binding protein (BiP2, spot 872) was decreased in *jin1-9/myc2* plants compared to WT plants after MeJA treatment (Table 1). The expression of BiP responds to multiple abiotic and biotic stressful conditions, such as fungus infestation, insect attack, nutrient deficiency, drought tolerance, cold stress and MeJA treatment [59,75-77]. The decreased induction of BiP2 in MeJA-treated *jin1-9/myc2* plants indicates that the plants might induce BiP to counteract abiotic and biotic stress in a MYC2-dependent manner in the JA signaling pathway.

### Photosynthesis-related proteins

We identified six photosynthesis-related proteins, including two whose expression was increased and four whose expression was decreased (Table 1). With regards to proteins involved in chlorophyll biosynthesis, there was a decrease in the induction of coproporphyrinogen III oxidase (spot 233) and uroporphyrinogen decarboxylase 2 (spot 247) after 48 h of MeJA treatment in *jin1-9/myc2* plants compared to WT plants. Regarding light reaction-related proteins, the expression of a chlorophyll a/b-binding protein (spot 126) was increased in *jin1-9/myc2* plants after 6 h of MeJA treatment. In addition, the expression of ribulose bisphosphate carboxylase (Rubisco, spot 346), a Calvin cycle-related protein, was decreased in *jin1-9/myc2* plants after 48 h of MeJA treatment, whereas transketolase-like protein (spot 865) was increased in *jin1-9/myc2* plants after 6 h of MeJA treatment (Table 1). JA has an inhibitory effect on photosynthesis [78] because it represses the expression of photosynthesis-related genes [79,80]. Chlorophyll biosynthesis-related proteins, chlorophyll a/b-binding proteins, and Rubisco proteins were down-regulated by MeJA treatment [59], and treatment with exogenous JA or MeJA caused a decrease in the rate of photosynthetic CO_2_ fixation, Rubisco activity, and chlorophyll content [80,81]. Rubisco is the major enzyme that limits photosynthetic CO_2_ assimilation and photorespiration [82], while transketolase plays a key role in limiting the maximum rate of photosynthesis in the presence of saturating light and CO_2_[83]. The increase in the level of chlorophyll a/b-binding protein and transketolase-like protein after 6 h of MeJA treatment in *jin1-9/myc2* plants compared to WT indicates that there is likely a temporary increase in the photosynthetic rate in MeJA-treated *jin1-9/myc2* plants. However, the decreased induction of Rubisco, coproporphyrinogen III oxidase, and uroporphyrinogen decarboxylase 2 expression in *jin1-9/myc2* plants compared to WT plants suggests that the photosynthetic rate might be decreased in long-term MeJA-treated *jin1-9/myc2* plants. Thus, MYC2 could be mediating diverse roles during different stages of the JA signaling pathway. It has been reported that JA-regulated posttranscriptional expression of Rubisco and transketolase requires the inhibitory effect of COI1 [46]. Considering the diverse roles of MYC2 observed in our study, we speculate that JA probably inhibits photosynthesis via the COI1-MYC2 pathway.

### Carbohydrate metabolism-related proteins

It has been reported that MeJA can induce carbohydrate catabolism [59]. During carbohydrate metabolism, carbohydrates are hydrolyzed to monosaccharides, which subsequently undergo glycolysis, the tricarboxylic acid cycle (TCA cycle) and electron transport chain/oxidative phosphorylation under ideal conditions. 3-Oxoacyl-[acyl-carrier protein] reductase (NADH, spot 272) and 2-oxoglutarate dehydrogenase, E3 subunit (E3, spot 766), which are commonly involved in the TCA cycle, are decreased in abundance following MeJA treatment in *jin1-9/myc2* plants (Table 1). Several proteins involved in the TCA cycle, including the E3, were increased by MeJA treatment [59], and sucrose levels were reduced in MeJA-treated *Medicago truncatula* cell culture samples [84]. Based on these published observations and our results, we believe that MYC2 has a positive role in MeJA-induced carbohydrate metabolism.

### Protein synthesis, folding, and degradation-related proteins

MeJA leads to the suppression of total protein and RNA synthesis as well as the activity of nuclear RNA polymerases [85]. 40S ribosomal protein S12-1 (spot 38), TCP-1/cpn60 chaperonin family protein (spot 799) and ATP-dependent Clp protease proteolytic subunit 2 (spot 178) were decreased in MeJA-treated *jin1-9/myc2* plants compared to WT plants (Table 1). Protein folding and degradation play a vital role in the regulation of metabolic processes and stress responses. The key rate-limiting enzymes and misfolded/damaged proteins are regulated by different strategies in plants. Correct folding and subsequent assembly into oligomers is required for functional enzymes. Plants can refold the misfolded proteins by chaperonins, which includes the TCP-1/cpn60 chaperonin protein family [86-88]. It was reported that the TCP-1/cpn60 chaperonin proteins are increased in response to oxygen radicals and bacterial infection and are essential for the correct folding and assembly of polypeptides into oligomeric structures [86,89]. Further, plants can remove the excess or the misfolded/damaged proteins by proteolysis, and most of the targeted intracellular proteolysis is performed by the energy-dependent Clp protease [90]. These results imply that MYC2 is a positive mediator of JA-regulated protein folding and degradation.

## Conclusions

**Figure 4:**
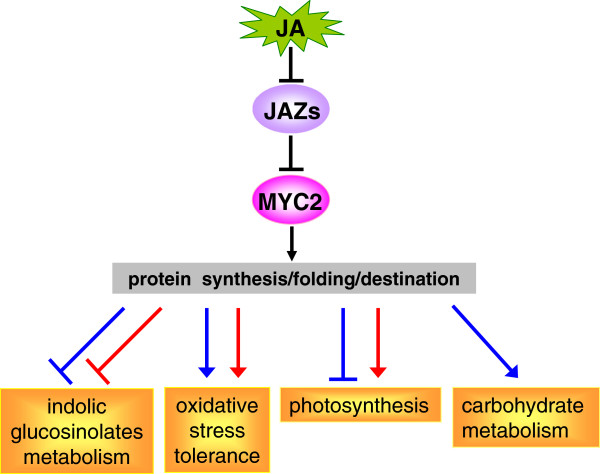
**MYC2 differentially regulates different JA-dependent biological process.** The role of MYC2 is indicated in blue and red to specify 6 h and 48 h MeJA treatments, respectively. Arrows and blunt arrows indicate positive and negative regulation, respectively.

In this study, we applied comparative proteomic approaches to obtain a comprehensive proteomic description of MeJA-treated *jin1-9/myc2* and WT plants. Quantitative analysis of 1500 proteins on 2D gels showed that 30 protein spots changed significantly, and of these, 27 were identified by MS analysis, and their functions were determined. The identified proteins were involved in glucosinolate metabolism, stress and defense, photosynthesis, carbohydrate metabolism, protein folding, and degradation, indicating that MYC2 regulates many of the JA-dependent functions in Arabidopsis. We observed that MYC2 exerts negative and positive effects on indolic glucosinolate biosynthetic enzymes and the myrosinase system, respectively. These results imply that MYC2 is a negative regulator of the JA-dependent accumulation of indolic glucosinolates. Interestingly, we observed that MYC2 exerts opposite effects on the biosynthetic enzymes of indolic and aliphatic glucosinolate pathways, and this is likely due to the homeostatic control of glucosinolate synthesis. This hypothesis needs to be further studied. Most of the MeJA-inducible proteins that are involved in multiple protective systems against oxidative stress were reduced in *jin1-9/myc2* plants compared to WT plants, consistent with a positive role for MYC2 in regulating JA-mediated oxidative stress tolerance. In addition, it was interesting to note that JA-responsive proteins that are implicated in chlorophyll biosynthesis, light reaction, and the Calvin cycle were increased at 6 h but decreased at 48 h post-MeJA treatment in *jin1-9/myc2* plants compared to WT plants. These results imply that MYC2 mediates diverse roles in the modulation of a subset of JA-regulated photosynthesis-related proteins. Moreover, a decrease in the expression of proteins involved in carbohydrate metabolism in 6 h MeJA-treated *jin1-9/myc2* plants compared to WT plants suggests that MYC2 positively regulates MeJA-induced carbohydrate metabolism. A schematic diagram summarizing these findings is shown in Figure 4. Arabidopsis cells perceive JA signals and transmit them to MYC2 to regulate protein synthesis, folding, and degradation; therefore, MYC2 exerts a positive or negative effect on the levels of functional proteins involved in indolic and aliphatic glucosinolate metabolism, oxidative stress tolerance, photosynthesis, and carbohydrate metabolism. This model allows us to further understand the functions of MYC2 in coordinating JA-mediated responses in Arabidopsis.

## Materials and methods

### Plant material and treatment

Seeds of Arabidopsis ecotype Columbia (Col-0) and mutant line SALK_017005 (*jin1-9*) were obtained from the Arabidopsis Biological Resource Center. The location of the T-DNA insertion was verified using a nested PCR approach, and homozygous plants were used in all of the subsequent experiments. Seeds were sown in plastic trays filled with soil and vermiculite mixture (1:1). Seedlings were grown in a growth chamber with an 8 h light/16 h dark cycle, 25°C/20°C day/night temperature, 150 μmol m^-2^ s^-1^ light intensity and a relative humidity of 60%. Two-week-old seedlings (normally with six leaves) were then transferred to hydroponic culture. Fresh half-strength Murashige and Skoog (MS) medium [91] was added every 3 days. Seven days after transfer to hydroponic medium, the seedlings were treated with 200 μM MeJA by adding 4 mM stock solution of MeJA (also containing 2% of ethanol and 0.06% of Tween 20) or an equal volume of ethanol and Tween-20 to serve as mock controls, according to the protocol of Shan et al. [46]. Leaf samples were directly harvested into liquid nitrogen after 6 h and 48 h of treatment.

### Protein extraction

Approximately 1 g of fresh leaves was harvested from control and 200 μM MeJA-treated Col-0 and *jin1-9/myc2* mutants and ground into fine powder in liquid nitrogen. The powder was precipitated in a 10% (w/v) TCA and acetone solution containing 0.07% (v/v) β-mercaptoethanol at −20°C for at least 2 h. The mixture was centrifuged at 40,000 g at 4°C for 1 h, and the precipitates were washed with cold acetone that contained 0.07% (v/v) β-mercaptoethanol, 1 mM phenylmethylsulfonyl fluoride (PMSF), and 2 mM EDTA. Pellets were dried by vacuum centrifugation and dissolved in 7 M urea, 2 M thiourea, 4% (w/v) cholamidopropyl CHAPS, 20 mM dithiothreitol (DTT), 2% (v/v) pharmalyte 4–7 (Amersham Pharmacia Biotech, Uppsala, Sweden), and 1% (v/v) proteinase inhibitor (Amersham Pharmacia Biotech) at room temperature for 1 h before being centrifuged at 40,000 g at 4°C for 1 h. The supernatant was collected, and the protein concentration was determined using the 2-D Quant kit (GE Healthcare, USA) using BSA as a standard. Samples were frozen in liquid nitrogen and kept at −80°C until further use.

### 2-DE and data analysis

Immobiline Dry Strips (pH 4–7 linear, 24 cm long) were run at 30 V for 8 h, 50 V for 4 h, 100 V for 1 h, 300 V for 1 h, 500 V for 1 h, 1,000 V for 1 h, and 8,000 V for 12 h using the hydration buffer (8 M urea, 2% CHAPS, 20 mM DTT) containing 0.6% v/v IPG buffer. SDS-PAGE was performed using 12.5% polyacrylamide gels without a stacking gel. Proteins were visualized by Coomassie Brilliant Blue R250 staining, and gel images were acquired using an ImageScanner (GE Healthcare). Image analysis was performed with ImageMaster 2D Platinum Software Version 7.0 (Amersham Biosciences, Piscataway, NJ). In order to obtain reliable results from 2-DE images, protein samples were always prepared in triplicate. After automated detection and matching, manual editing was carried out to correct the mismatched and unmatched spots. Spots were considered reproducible if they appeared well-resolved in the three biological replicates. For each spot that matched, a measurement was carried out for each biological replicate, and normalized volumes were computed using the software’s total spot volume normalization procedure. The normalized volume of each spot was assumed to represent its expression abundance. The criteria used to define significant differences when analyzing parallel spots between groups with two-way ANOVA included a *p* < 0.05 and an abundance ratio of at least 1.5.

### In-gel digestion and protein identification

Protein digestion was performed as described previously [59]. For MALDI-TOF/TOF MS analysis, tryptic peptides were desalted with C18 Ziptips (Millipore) and spotted onto a MALDI plate by mixing 1:1 with the matrix solution (10 mg/mL CHCA in 60% ACN and 0.1% TFA). MS/MS spectra were acquired using a 4700 MALDI-TOF/TOF mass spectrometer (Applied Biosystems/MDS Sciex, USA). The peptide MS/MS spectra were searched against NCBI non-redundant fasta database (8,224,370 entries, downloaded on April 14, 2009) using the Mascot search engine (http://www.matrixscience.com). Mascot was set up to search green plants only, assume trypsin digestion and allow for one miscleavage. The mass tolerance for both parent ion and fragment ion mass was set to be 0.3 Da. Iodoacetamide derivatization of Cys, deamidation of Asn and Gln, and oxidation of Met are specified as variable modifications. Unambiguous identification was performed based on the number of peptides obtained, sequence coverage, MASCOT mowse score and the quality of MS/MS spectra.

### Quantitative real-time PCR (qRT-PCR)

The total RNA and qRT-PCR experiments were performed according to Guo et al. [92]. Reverse transcription was performed using the PrimeScript RT reagent Kit (TaKaRa, China). Triplicate quantitative assays were performed with 1 μL of cDNA (1:10 dilution) and the SYBR Green Master mix (TaKaRa) using an ABI 7500 sequence detection system (Applied Biosystems, USA). RNA expression was calculated based on a relative standard curve representing 5-fold dilutions of cDNA. The amplification of *ACTIN2* was used as an internal control and to normalize the data. The details of primers are listed in Additional file 3. The data analysis was performed using three technical replicates from one biological sample. Similar results were obtained with two other biological replicates.

## Abbreviations

2-DE: Two-dimensional gel electrophoresis; AMI1: Amidase 1; Arabidopsis: *Arabidopsis thaliana*; bHLH: Basic helix-loop-helix; COI1: CORONATINE-INSENSITIVE1; DTT: Dithiothreitol; E3: 2-oxoglutarate dehydrogenase, E3 subunit; GSH: Glutathione; GSTF10: Glutathione S-transferase ERD13; JA: Jasmonate; JAZ: JA ZIM-domain; JIN1: JASMONATE-INSENSITIVE1; MALDI: Matrix-assisted laser desorption/ionization; MBP: Myrosinase-binding protein; MeJA: Methyl jasmonate; MS: Murashige and Skoog; PMSF: Phenylmethylsulfonyl fluoride; qRT-PCR: Quantitative real-time PCR; ROS: Reactive oxygen species; Rubisco: Ribulose bisphosphate carboxylase; TCA cycle: Tricarboxylic acid cycle; TF: Transcription factor; TGG: Thioglucoside glucohydrolase; TOF: Time-of-flight; WT: Wild type.

## Competing interests

The authors declare that they have no competing interests.

## Authors’ contributions

JG conceived the study, prepared the samples, performed the qRT-PCR analysis and drafted the manuscript; QP performed the experiments and data acquisition and drafted the manuscript; LW, PY and NL assisted in performing the experiments; XY contributed to the overall design of this study. JG, QP and XY read and approved the final manuscript.

## Supplementary Material

Additional file 1 Peptide sequences and observed m/z of the identified proteins in MALDI-TOF/TOF mass spectrometry analysis. Click here for file

Additional file 2 **Protein identities and changes in their expression in*jin1-9/myc2*plants compared to wild-type plants after 6 h/48 h of mock treatment and between untreated*jin1-9/myc2*and wild-type plant.** Click here for file

Additional file 3 Sequences of the primers used for quantitative real-time PCR. Click here for file
